# Effects of Acute Exposure to Thermal Stress on Cardiorespiratory Function, Skeletal Muscle Oxygenation, and Exercise Performance in Healthy Males

**DOI:** 10.3390/ijerph18147404

**Published:** 2021-07-11

**Authors:** Won-Sang Jung, Sung-Woo Kim, Hun-Young Park, Jisu Kim, Kiwon Lim

**Affiliations:** 1 Physical Activity and Performance Institute, Konkuk University, 120 Neungdong-ro, Gwangjin-gu, Seoul 05029, Korea; jws1197@konkuk.ac.kr (W.-S.J.); kswrha@konkuk.ac.kr (S.-W.K.); parkhy1980@konkuk.ac.kr (H.-Y.P.); kimpro@konkuk.ac.kr (J.K.); 2 Department of Sports Medicine and Science, Graduate School, Konkuk University, 120 Neungdong-ro, Gwangjin-gu, Seoul 05029, Korea; 3 Department of Physical Education, Konkuk University, 120 Neungdong-ro, Gwangjin-gu, Seoul 05029, Korea

**Keywords:** thermal stress, cardiorespiratory function, skeletal muscle oxygenation, exercise performance, healthy males

## Abstract

We investigated the effects of acute thermal stress (30 °C and 40 °C) and ordinary temperature (20 °C) on cardiorespiratory function, skeletal muscle oxygenation, and exercise performance in healthy men. Eleven healthy males (21.5 ± 2.3 years) performed a graded exercise test (GXT) using a cycle ergometer in each environmental condition (20 °C, 30 °C, and 40 °C) in a random order with an interval of 1 week between each test. Before the test, they were allowed to rest for 30 min in a given environmental condition. All dependent variables (body temperature, cardiorespiratory function parameters, skeletal muscle oxygenation profiles, and exercise performance) were measured at rest and during GXT. GXT was started at 50 W and increased by 25 W every 2 min until subjects were exhausted. Body temperature increased proportionally at rest and at the end of exercise as thermal stress increased. There were no differences in the rating of perceived exertion, oxygen uptake, respiratory exchange ratio, and carbon dioxide excretion between environmental conditions. Heart rate (HR), minute ventilation (VE), and blood lactate levels were significantly higher at 30 °C and 40 °C than at 20 °C, and oxygen pulse was significantly lower at 40 °C than at 20 °C at various exercise loads. None of the skeletal muscle oxygenation profiles showed significant changes at rest or during exercise. Maximal oxygen uptake, peak power, and exercise time significantly decreased proportionally as thermal stress increased, and this decrease was most pronounced at 40 °C. Acute thermal stress induces a decrease in exercise performance via increased body temperature, HR, VE, and blood lactate levels and decreased oxygen pulse during load-homogenized exercise. This phenomenon was more prominent at 40 °C than at 30 °C and 20 °C.

## 1. Introduction

Depending on the nature of the sport they play, athletes are often in situations where they have to perform under thermal stress [1]. Exercise under thermal stress causes more physical strain to athletes than does exercise under normal conditions, and results in dehydration and cardiovascular strain, thereby reducing exercise performance [2]. Increase in body temperature due to prolonged thermal stress highly correlates with the cardiovascular system, neuromuscular system, metabolism, immunology, and perception (thermal sensation, thermal comfort, and rating of perceived exertion (RPE)). Exercise under thermal stress causes fatigue due to interactions between cardiovascular, respiratory, and central and peripheral neuromuscular system factors [3,4]. Central and peripheral fatigue, important determinants of exercise performance under acute exposure to thermal stress, appear along with changes in body temperature, heart rate (HR), inflammation, and stress indices, which subsequently increase RPE during submaximal exercise and exacerbates exercise performance [5,6,7]. Consequently, a shorter time to exhaustion and longer trial completion time are generally associated with thermal stress [8,9,10,11,12].

Acute exposure to thermal stress also leads to various physiological responses in the metabolic and cardiovascular systems [10,12,13,14]. Exercise under thermal stress increases the activity of the sympathetic nervous system (SNS) and decreases the activity of the parasympathetic nervous system [15]. It also increases the resistance of peripheral blood vessels to meet the increased metabolic rate in skeletal muscle and to increase blood flow to the skin. This reduces body temperature, thereby increasing HR, blood pressure, and cardiac output [11]. In addition, the increase in the activity of the SNS causes an upregulation in the secretion of catecholamine, which promotes the breakdown of muscle glycogen and glycolysis, thereby increasing the levels of metabolites and lactate in the blood [10,14]. Exercise under acute thermal stress limits oxygen delivery and utilization capacity of the muscle tissue through the physiological mechanisms mentioned above, thereby reducing the anaerobic threshold and maximal oxygen uptake (VO_2_max) and attenuating exercise performance [10,12,14].

Acute thermal stress can reduce exercise performance by inducing various physiological responses including neuromuscular, metabolic, and cardiovascular functions. The traditional belief was that skeletal muscle blood flow is not changed by thermal stress and that increases in cardiac output support elevated skin blood flow requirements [16,17]. However, Chiesa et al. [18], Pearson et al. [19], and Gonzalez-Alonso et al. [20] reported that elevated tissue/blood temperatures via thermal stress induce an increase in skeletal muscle blood flow during rest and exercise, and Gonzalez-Alonso et al. [20] reported that the mechanisms mediating this increase in skeletal muscle blood flow may include an interaction of metabolic and thermal stimuli that induce the release of erythrocyte-derived ATP, a potent vasodilator. Thermal stress caused large increase in the tissue oxygenation signal and these increases were closely correlation with increases in skin blood flow, and the role of local oxygen and peripheral circulation in thermal stress and their connection to exercise performance is still not explained. However, previous studies examining the relationship between thermal stress and exercise performance only verified the effects of light thermal stress (30–35 °C) and ordinary temperature (20–23 °C) on HR and metabolic response by respiratory gas analysis [10,14,21].

Therefore, it is important to utilize near-infrared spectroscopy (NIRS), which can examine the local oxygen availability in muscle tissue, to accurately identify the relationship between thermal stress and exercise performance [1,22,23]. In addition, since local oxygenation and peripheral circulation play an important role in blood flow and oxygen supply to muscle tissue, the use of NIRS can provide a basis for interpreting the cause of the decrease in exercise performance due to thermal stress [1].

We hypothesized that acute thermal stress would affect exercise performance via changes in the metabolic response and respiratory/circulatory system as well as changes in skeletal muscle oxygenation that can be measured using NIRS. Based on this hypothesis, the present study aimed to investigate the effect of acute thermal stress (30 °C and 40 °C) vs. ordinary temperature (20 °C) on cardiorespiratory function, skeletal muscle oxygenation, and exercise performance in healthy males.

## 2. Materials and Methods

### 2.1. Subjects

The subjects in this study were healthy males (*n* = 11) with no history of smoking and orthopedic or medical diseases in the past year. The physical characteristics of the subjects are shown in Table 1. A total of three graded exercise tests (GXTs) under various thermal stress conditions (30 °C and 40 °C) and at ordinary temperature (20 °C) were conducted. Environmental conditions were randomly selected, and each condition was set up with a wash-out period of 2 weeks. We explained the experiments and possible side effects to all subjects prior to the start of the study and obtained written consent for participation. This study was approved by the Institutional Review Board of Konkuk University (7001355-201805-HR-241) in Korea and was conducted in accordance with the provisions of the Declaration of Helsinki.

### 2.2. Study Design

**Figure 1:**
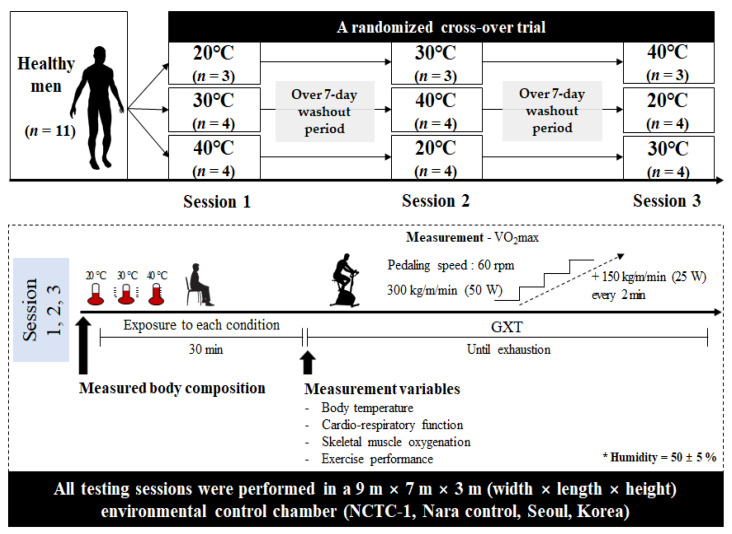
Study design. VO_2_max, maximal oxygen uptake.

**Figure 2:**
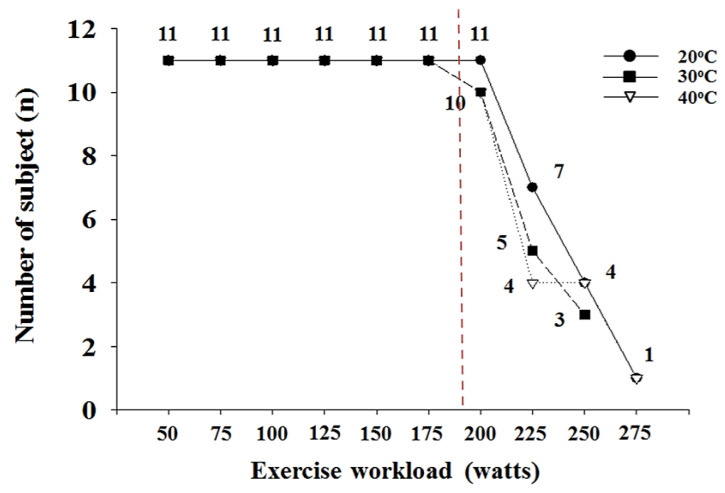
The end of graded exercise test at each environmental condition in all subjects.

The study design is illustrated in Figure 1. All subjects visited our laboratory four times during the experimental period. Subjects’ body composition was measured during the first visit. To minimize the adaptation effect, thermal stress conditions (30 °C and 40 °C) and ordinary temperature condition (20 °C) were randomly selected, and each environmental condition had a wash-out period of at least 1 week. During the experiment, all subjects were allowed to rest for 30 min in each environmental condition, and then the dependent parameters, including body temperature, cardiorespiratory function, skeletal muscle oxygenation, and exercise performance, were measured. All subjects performed GXTs in each environmental condition using a cycle ergometer (Monark ergomedic 828E, Monark, Varberg, Sweden). The GXT protocol was started at 300 kg·m·min^−1^ (50 W), and the exercise load was increased by 150 kg·m·min^−1^ (25 W) every 2 min; the pedaling speed was set to 60 rpm. For data acquisition during exercise, cardiorespiratory function parameters and skeletal muscle oxygenation profiles were measured in increments of 10 s, and the average value for every 2 min in each section was calculated as the measured value. Data processing for dependent parameters related to cardiorespiratory function and skeletal muscle oxygenation profiles at each exercise intensity during GXT was performed up to 175 W, the intensity at which all subjects performed GXT (Figure 2). Data after 175 W were used only to measure and evaluate exercise performance. Blood lactate level was measured every minute during GXT, and the average value for every 2 min in each section was used. Body temperature was measured at rest and immediately after GXT. All testing sessions were performed in a 9 m × 7 m × 3 m (width × length × height) environmental control chamber (NCTC-1, Nara Control, Seoul, Korea). The chamber was used to create thermal stress conditions (30 °C and 40 °C) and ordinary temperature condition (20 °C). Humidity was maintained at 50 ± 5% in all the different environmental conditions.

### 2.3. Anthropometric and Body Composition

Height was measured using a stadiometer (YM-1, KDS, Seoul, Korea) and body composition parameters (weight, muscle mass, and percent body fat) were analyzed using a bioelectrical impedance analyzer (Inbody 770; Inbody, Seoul, Korea). BMI was measured by the following formula: weight (kg)/height^2^ (m^2^). All subjects wore lightweight clothing and were asked to remove any metal items.

### 2.4. Graded Exercise Test and Measurements of Dependent Variables

GXTs were performed under thermal stress conditions (30 °C and 40 °C) and an ordinary temperature (20 °C) using a cycle ergometer (Monark ergomedic 828E, Monark, Varberg, Sweden). The GXT protocol was started at 300 kg·m·min^−1^ (50 W), and the exercise load was increased by 150 kg·m·min^−1^ (25 W) every 2 min, and the pedaling speed was set to 60 rpm. The tests were started after subjects wore a mask and all respiratory gas parameters were stabilized, and VO_2_max was measured using an automatic breathing gas analyzer K5 (COSMED, Monte Savello, Italy). When at least three of the following conditions were satisfied, a subject was judged to have reached the stage of exhaustion, and GXT was terminated: 1. When HR did not increase in proportion to exercise intensity; 2. When VO_2_ did not increase, even when exercise intensity increased; 3. When respiratory exchange ratio (RER) was 1.10 or higher; 4. When Borg scale score was 17 or higher; and 5. When the predicted HRmax exceeded 90%.

During the GXT in each environmental condition, body temperature, cardiorespiratory function parameters, skeletal muscle oxygenation profiles, and exercise performance were evaluated. Body temperature was measured in the ear, at rest, and immediately after GXT using an IRT-6510 thermometer (Brown Healthcare, Seoul, Korea). Measurements were taken twice, and the average value was used.

Cardiorespiratory function parameters were measured at rest and during exercise via breathing gas using an automatic respiration metabolic analyzer K5 (COSMED, Monte Savello, Italy), Monark cycle ergometer (Monark ergomedic 828E, Monark, Varberg, Sweden), and HR monitor (V800, Polar, Helsinki, Finland). The cardiorespiratory function parameters, RPE, HR, minute ventilation (VE), oxygen uptake (VO_2_), oxygen pulse, carbon dioxide excretion (VCO_2_), and RER were measured in increments of 10 s, and the average value for every 2 min in each section was used. RPE was measured every 2 min using the Borg scale. Blood lactate levels were analyzed using a Lactate Pro2 analyzer (Arkray, Kyoto, Japan) by collecting 80 μL of blood from capillaries at the fingertips every minute at rest and during GXT, and the average value for every 2 min in each section was calculated as the measured value.

Skeletal muscle oxygenation profiles, including oxygenated hemoglobin level (Oxy_Hb), deoxygenated hemoglobin level (Deoxy_Hb), total hemoglobin level (Total_Hb), and tissue oxygen saturation (S_t_O_2_), were measured using an NIRS system for muscle tissue (Astem, Mizonokuchi, Japan) in the right vastus lateralis muscle. After attaching the NIRS probe to the vastus lateralis muscle 10–15 cm above the knee, the data were recorded every 10 s from rest to the end of GXT, and an average value of 2 min was used.

To evaluate the difference in exercise performance between thermal stress conditions (30 °C and 40 °C) and ordinary temperature (20 °C), VO_2_max, peak power, and exercise time to exhaustion during GXT were measured.

### 2.5. Statistical Analysis

The means and standard deviations were calculated for each primary dependent parameter. The normality and homoscedasticity of all outcome parameters were verified using a Shapiro–Wilk test. A two-way analysis of variance with repeated measures of the “time” and “environmental condition” factors was used to analyze the effects of interaction or the main effect on each dependent variable. If a significant interaction or main effect was found, a one-way analysis of variance with repeated measures was used to verify differences according to time or environmental conditions, and a Bonferroni post hoc test was used. The level of significance was set a priori at 0.05.

## 3. Results

### 3.1. Body Temperature

**Figure 3:**
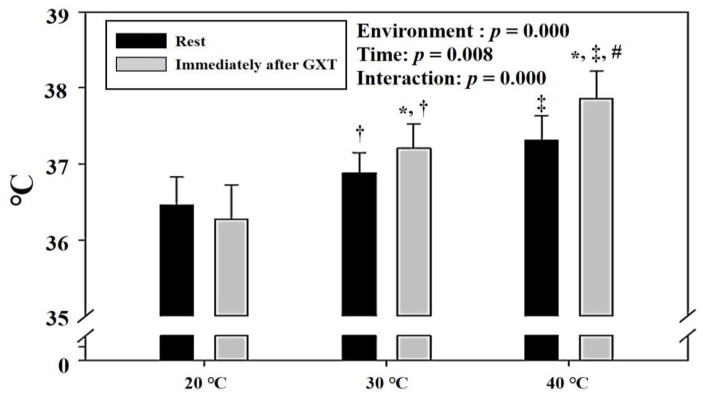
Changes in body temperature at rest and immediately after GXT (mean ± standard deviation). GXT: graded exercise test; a: significant difference immediately after GXT; *: significant difference between rest and immediately after GXT; †: significant difference between 20 °C and 30 °C; ‡: significant difference between 20 °C and 40 °C; #: significant difference between 30 °C and 40 °C.

Figure 3 shows the change in body temperature at rest and immediately after GXT under various thermal stress conditions (30 °C and 40 °C) and ordinary temperature (20 °C). There was a significant interaction between environmental conditions and time (*p* < 0.001). Post hoc analysis showed that there was a significant increase in body temperature at 30 °C and 40 °C immediately after GXT compared with that at rest (*p* < 0.05). In addition, body temperature showed a significant proportional increase at rest and immediately after GXT as the thermal stress increased (*p* < 0.05).

### 3.2. Cardiorespiratory Function

**Figure 4:**
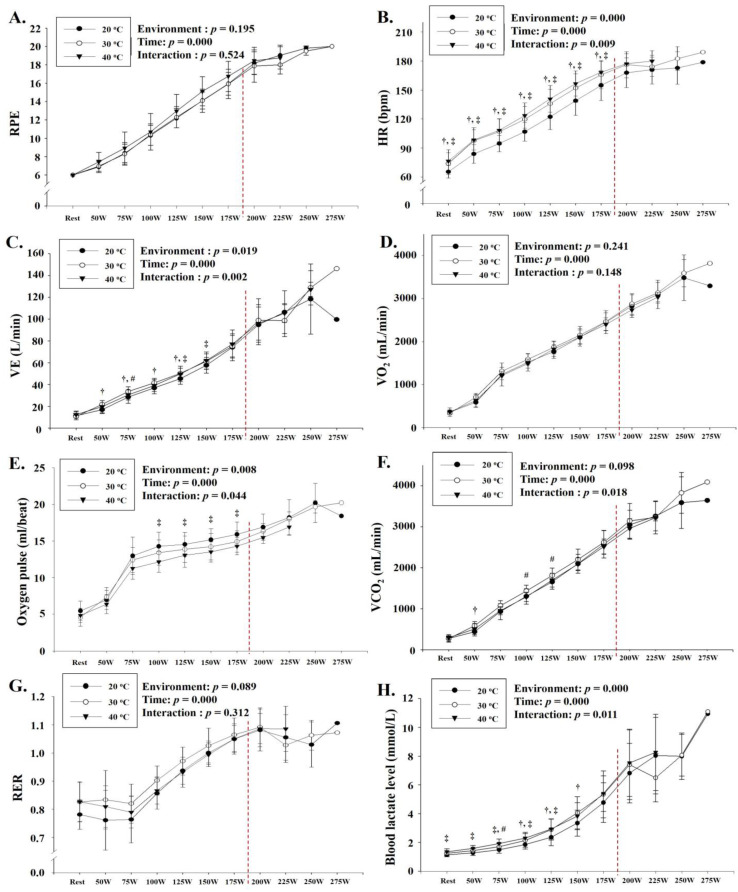
Changes in cardiorespiratory function parameters at rest and during GXT (mean ± standard deviation): (**A**) change in RPE, (**B**) change in HR, (**C**) change in VE, (**D**) change in VO_2_, (**E**) change in oxygen pulse, (**F**) change in VCO_2_, (**G**) change in RER, and (**H**) change in blood lactate level are shown. GXT: graded exercise test; RPE: rating of perceived exertion; HR: heart rate; VE: minute ventilation; VO_2_: oxygen uptake; VCO_2_: carbon dioxide excretion; RER: respiratory exchange ratio. †: significant difference between 20 °C and 30 °C; ‡: significant difference between 20 °C and 40 °C; #: significant difference between 30 °C and 40 °C.

As shown in Figure 4, there was a significant interaction between environmental conditions and time in HR (*p* = 0.009), VE (*p* = 0.002), oxygen pulse (*p* = 0.044), VCO_2_ (*p* = 0.018), and blood lactate level (*p* = 0.011). Post hoc analysis revealed that HR, VE, and blood lactate levels showed a significant increase at almost all exercise loads as thermal stress increased (*p* < 0.05). Oxygen pulse was significantly lower at 40 °C than at 20 °C (*p* < 0.05). VCO_2_ showed a significant decrease at 40 °C compared with that at 30 °C and 20 °C (*p* < 0.05). No significant interaction between environmental conditions and time was observed for RPE, VO_2_, and RER.

### 3.3. Skeletal Muscle Oxygenation

**Figure 5:**
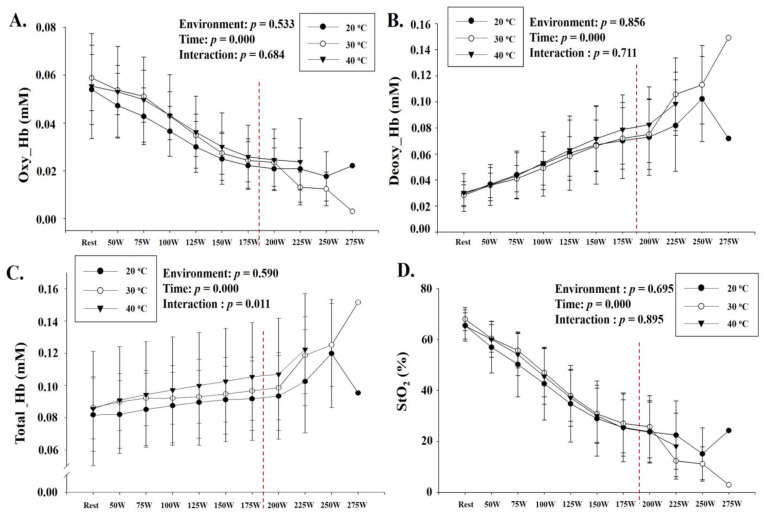
Changes in skeletal muscle oxygen profiles at rest and during GXT (mean ± standard deviation): (**A**) change in Oxy_Hb, (**B**) change in Deoxy_Hb, (**C**) change in Total_Hb, and (**D**) change in S_t_O_2_ are shown. GXT: graded exercise test; Oxy_Hb: oxygenated hemoglobin; Deoxy_Hb: deoxygenated hemoglobin; Total_Hb: total hemoglobin; S_t_O_2_: tissue oxygen saturation.

Figure 5 depicts the changes in skeletal muscle oxygenation profiles at rest and during GXT under various thermal stress conditions (30 °C and 40 °C) and ordinary temperature (20 °C). There was a significant interaction between environmental conditions and time in Total_Hb (*p* = 0.011), but post hoc analysis did not show any difference in Total_Hb between environmental conditions at each time point. No significant interaction between environmental conditions and time was observed for Oxy_Hb, Deoxy_Hb, and S_t_O_2_.

### 3.4. Exercise Performance

**Figure 6:**
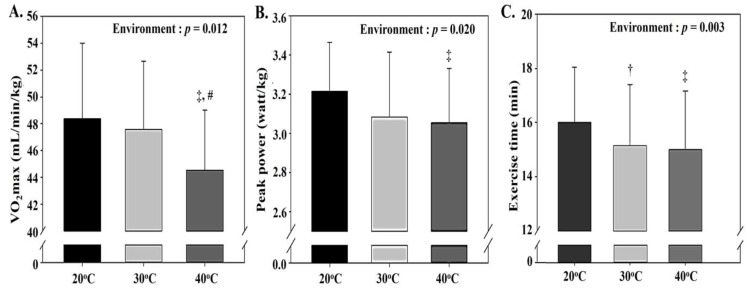
Changes in exercise performance parameters (mean ± standard deviation): (**A**) change in VO_2_max, (**B**) change in peak power, and (**C**) change in exercise time to exhaustion are shown. GXT: graded exercise test; VO_2_max: maximal oxygen uptake. †: significant difference between 20 °C and 30 °C; ‡: significant difference between 20 °C and 40 °C; #: significant difference between 30 °C and 40 °C.

As depicted in Figure 6, there was a significant effect of environmental conditions on VO_2_max (*p* = 0.012), peak power (*p* = 0.020), and exercise time (*p* = 0.003). Post hoc analysis revealed that VO_2_max showed a significant decrease at 40 °C compared with that at 30 °C and 20 °C (*p* < 0.05). Peak power was significantly lower at 40 °C than at 20 °C (*p* < 0.05). Exercise time showed a significant proportional decrease with an increase in thermal stress (*p* < 0.05).

## 4. Discussion

This study was based on the hypothesis that acute thermal stress would affect exercise performance via changes in the cardiorespiratory function as well as through changes in skeletal muscle oxygenation that can be measured using NIRS. Our study demonstrated that acute thermal stress (30 °C or 40 °C) vs. ordinary temperature (20 °C) attenuates exercise performance via increased body temperature, HR, VE, and blood lactate levels and decreased oxygen pulse during a load-homogenized exercise. It was confirmed that this phenomenon was more prominent at 40 °C than at 30 °C. However, contrary to our hypothesis, there was no significant difference in the skeletal muscle oxygenation profiles (Oxy_Hb, Deoxy_Hb, Total_Hb, or S_t_O_2_), measured using NIRS, under various thermal stress conditions.

Various previous studies reported that acute thermal stress attenuates the speed and strength of the human body during exercise and affects various physiological responses and, therefore, the exercise performance [3,9,10]. Exercise under thermal stress not only reduces exercise performance but also increases the risk of heat injury [14]. Previous laboratory-based studies have demonstrated reduced endurance of the exercise, measured by exercise time to exhaustion, under thermal stress [10,24]. In addition, thermal stress contributes to reduced marathon running performance, and a previous study suggested that marathon records deteriorated by 2–3% when wet-bulb globe temperature exceeded 20 °C [25,26]. Exercise under thermal stress induces fatigue and reduces exercise performance via several physiological and metabolic factors, including cardiorespiratory function, fluid balance, central nervous system function, and motor drive [14]. In terms of metabolic function, exercise under thermal stress activates the SNS to increase the secretion of catecholamine, which in turn activates glycolysis to increase muscle glycogen degradation and blood lactate levels [15,24,27,28]. With regard to cardiovascular function, the body temperature control mechanism under acute thermal stress is usually heat transfer to the skin via cutaneous vasodilation, loss of body fluid, and evaporation of sweat [14]. However, exercise under thermal stress induces vasoconstriction, which is reported to be caused by a circulatory conflict between the skin and activated skeletal muscle [29]. In particular, high-intensity or prolonged exercise under thermal stress increases HR at the same exercise intensity and decreases stroke volume, cardiac output, and muscle blood flow, thereby reducing VO_2_max and exercise performance [30,31]. This deterioration of cardiovascular function can reduce the blood flow to the active muscle, which can worsen the efficiency of oxygen supply and use in the tissues [29,30,31]. In the present study, VE and blood lactate levels showed a significant increase at almost all exercise loads as thermal stress increased (40 °C > 30 °C > 20 °C), and VCO_2_ showed a significant decrease at 40 °C compared with the values at 30 °C and 20 °C. This result is consistent with the results of previous studies showing that exercise under thermal stress activates anaerobic metabolism and increases dependence on glycogen via glycolysis and blood lactate levels [15,24,27,28]. Additionally, HR and oxygen pulse showed significant proportional decrease at almost all exercise loads as thermal stress increased, and this result is consistent with the findings of previous studies that, as described above, exercise under thermal stress attenuates the efficiency of the cardiorespiratory function [29,30,31]. However, given that the primary metabolic and cardiovascular function challenge during exercise in the thermal stress is to adequately perfuse skeletal muscle to support metabolism while providing sufficient cardiac output to perfuse the skin to support heat loss, the fact that stroke volume and cardiac output were not measured in the present study is a major limitation of the study.

NIRS is a noninvasive optical technique that is increasingly being used to assess changes in oxygen concentrations in skeletal muscle tissue [32]. Therefore, NIRS is widely used to examine local changes in skeletal muscle oxygenation and blood flow during maximal and submaximal exercise [33]. Davis et al. [34] tested the effects of locally applied heat and indirect whole-body heating on tissue oxygenation and reported that these two interventions increased the tissue oxygenation signal and that this increase was closely related to the increase in skin blood flow. Therefore, we hypothesized that thermal stress would affect blood flow in local tissues, that is, oxygen transport and utilization capacity, and deteriorate exercise performance. Therefore, we verified the effect of exercise under thermal stress on skeletal muscle oxygenation in the right latissimus lateral muscle using NIRS. However, there was no significant difference between the thermal stress conditions (30 °C and 40 °C) and ordinary temperature (20 °C) in Oxy_Hb, Deoxy_Hb, Total_Hb, and S_t_O_2_, which are parameters of skeletal muscle oxygenation. Périard et al. [35] examined the change in skeletal muscle oxygenation in the vastus lateralis during cycling to exhaustion under thermal stress conditions (40 °C) and ordinary temperature (18 °C) and reported that thermal stress decreased vastus lateralis oxygen saturation, which increases cardiovascular strain during exercise and attenuates exercise performance. The difference in results between our study and those of Périard et al. [35] is thought to be due to differences in study designs as we examined the effect of thermal stress on skeletal muscle oxygenation at the same exercise intensity during GXT, but they verified the effect of thermal stress on skeletal muscle oxygenation at a steady state (60% VO_2_max) for 60 min. As there are no previous studies examining the difference in skeletal muscle oxygenation concentration under the same load in GXT with the same experimental design as ours, future studies should comprehensively review the effects of thermal stress on cardiorespiratory function and skeletal muscle oxygenation to determine the exact mechanism.

All exercise performance parameters (VO_2_max, peak power, and exercise time to exhaustion) showed a significant decrease proportionate to the increase in thermal stress, and this decrease was most pronounced at 40 °C. As described above, the decrease in exercise performance via thermal stress is considered to be due to the increase in anaerobic metabolism (i.e., elevated blood lactate level and VE) and the decrease in cardiorespiratory function (i.e., increased HR and decreased oxygen pulse) [15,24,27,28,29,30,31].

## 5. Conclusions

Our study demonstrated that exercise under acute thermal stress attenuates exercise performance via increased body temperature, HR, VE, and blood lactate levels and decreased oxygen pulse during load-homogenized submaximal exercise in healthy adults. This physiological response to thermal stress was found to be more pronounced at 40 °C than at 30 °C and 20 °C (40 °C > 30 °C > 20 °C). However, skeletal muscle oxygenation measured using NIRS showed no significant changes due to thermal stress.

## Figures and Tables

**Table 1:** Characteristics of subjects.

Variables	Mean ± SD
Age (years)	21.5 ± 2.2
Height (cm)	178..3 ± 5.5
Weight (kg)	71.1 ± 4.2
BMI (kg/m^2^)	22.3 ± 1.3
Muscle mass (kg)	57.8 ± 4.8
Percent body fat (%)	13.5 ± 3.6

Values are expressed as mean ± SD. BMI, body mass index; SD, standard deviation.

## References

[1] Park H.-Y., Jung W.-S., Hwang H., Kim S.-W., Jung K., Park Y., Hwang D., Kyun S., Seo J., Ha Y. (2020). Effects of acute cold stress on energy metabolism, skeletal muscle oxygenation, and exercise performance. KJSS.

[2] Guy J.H., Deakin G.B., Edwards A.M., Miller C.M., Pyne D.B. (2015). Adaptation to hot environmental conditions: An exploration of the performance basis, procedures and future directions to optimise opportunities for elite athletes. Sports Med..

[3] Cheung S.S., Sleivert G.G. (2004). Multiple triggers for hyperthermic fatigue and exhaustion. Exerc. Sport Sci. Rev..

[4] Nybo L., Rasmussen P., Sawka M.N. (2014). Performance in the heat-physiological factors of importance for hyperthermia-induced fatigue. Compr. Physiol..

[5] Donovan K.A., Stein K.D., Lee M., Leach C.R., Ilozumba O., Jacobsen P.B. (2015). Systematic review of the multidimensional fatigue symptom inventory-short form. Support Care Cancer.

[6] Tamm M., Jakobson A., Havik M., Burk A., Timpmann S., Allik J., Oöpik V., Kreegipuu K. (2014). The compression of perceived time in a hot environment depends on physiological and psychological factors. Q. J. Exp. Psychol..

[7] Tamm M., Jakobson A., Havik M., Timpmann S., Burk A., Ööpik V., Allik J., Kreegipuu K. (2015). Effects of heat acclimation on time perception. Int. J. Psychophysiol..

[8] Galloway S.D., Maughan R.J. (1997). Effects of ambient temperature on the capacity to perform prolonged cycle exercise in man. Med. Sci. Sports Exerc..

[9] Girard O., Racinais S. (2014). Combining heat stress and moderate hypoxia reduces cycling time to exhaustion without modifying neuromuscular fatigue characteristics. Eur. J. Appl. Physiol..

[10] Tatterson A.J., Hahn A.G., Martin D.T., Febbraio M.A. (2000). Effects of heat stress on physiological responses and exercise performance in elite cyclists. J. Sci. Med. Sport.

[11] Périard J.D., Cramer M.N., Chapman P.G., Caillaud C., Thompson M.W. (2011). Neuromuscular function following prolonged intense self-paced exercise in hot climatic conditions. Eur. J. Appl. Physiol..

[12] Sawka M.N., Leon L.R., Montain S.J., Sonna L.A. (2011). Integrated physiological mechanisms of exercise performance, adaptation, and maladaptation to heat stress. Compr. Physiol..

[13] Narayan E.J., Hero J.M. (2014). Acute thermal stressor increases glucocorticoid response but minimizes testosterone and locomotor performance in the cane toad (Rhinella marina). PLoS ONE.

[14] Hargreaves M. (2008). Physiological limits to exercise performance in the heat. J. Sci. Med. Sport.

[15] Low D.A., Keller D.M., Wingo J.E., Brothers R.M., Crandall C.G. (2011). Sympathetic nerve activity and whole body heat stress in humans. J. Appl. Physiol..

[16] Rowell L.B. (1974). Human cardiovascular adjustments to exercise and thermal stress. Physiol. Rev..

[17] Johnson J.M. (1977). Regulation of skin circulation during prolonged exercise. Ann. N. Y. Acad. Sci..

[18] Chiesa S.T., Trangmar S.J., Kalsi K.K., Rakobowchuk M., Banker D.S., Lotlikar M.D., Ali L., González-Alonso J. (2015). Local temperature-sensitive mechanisms are important mediators of limb tissue hyperemia in the heat-stressed human at rest and during small muscle mass exercise. Am. J. Physiol. Heart Circ. Physiol..

[19] Pearson J., Low D.A., Stöhr E., Kalsi K., Ali L., Barker H., González-Alonso J. (2011). Hemodynamic responses to heat stress in the resting and exercising human leg: Insight into the effect of temperature on skeletal muscle blood flow. Am. J. Physiol. Regul. Integr. Comp. Physiol..

[20] González-Alonso J., Calbet J.A., Boushel R., Helge J.W., Søndergaard H., Munch-Andersen T., van Hall G., Mortensen S.P., Secher N.H. (2015). Blood temperature and perfusion to exercising and non-exercising human limbs. Exp. Physiol..

[21] No M., Kwak H.B. (2016). Effects of environmental temperature on physiological responses during submaximal and maximal exercises in soccer players. Integr. Med. Res..

[22] Binzoni T., Ngo L., Hiltbrand E., Springett R., Delpy D. (2002). Non-standard O(2) consumption-temperature curves during rest and isometric exercise in human skeletal muscle. Comp. Biochem. Physiol. A Mol. Integr. Physiol..

[23] Hom C., Vasquez P., Pozos R.S. (2004). Peripheral skin temperature effects on muscle oxygen levels. J. Therm. Biol..

[24] Parkin J.M., Carey M.F., Zhao S., Febbraio M.A. (1999). Effect of ambient temperature on human skeletal muscle metabolism during fatiguing submaximal exercise. J. Appl. Physiol..

[25] Trapasso L.M., Cooper J.D. (1989). Record performances at the Boston Marathon: Biometeorological factors. Int. J. Biometeorol..

[26] Ely M.R., Cheuvront S.N., Roberts W.O., Montain S.J. (2007). Impact of weather on marathon-running performance. Med. Sci. Sports Exerc..

[27] Febbraio M.A., Snow R.J., Hargreaves M., Stathis C.G., Martin I.K., Carey M.F. (1994). Muscle metabolism during exercise and heat stress in trained men: Effect of acclimation. J. Appl. Physiol..

[28] Febbraio M.A. (2001). Alterations in energy metabolism during exercise and heat stress. Sports Med..

[29] Nielsen B., Savard G., Richter E.A., Hargreaves M., Saltin B. (1990). Muscle blood flow and muscle metabolism during exercise and heat stress. J. Appl. Physiol..

[30] González-Alonso J., Calbet J.A. (2003). Reductions in systemic and skeletal muscle blood flow and oxygen delivery limit maximal aerobic capacity in humans. Circulation.

[31] González-Alonso J., Calbet J.A., Nielsen B. (1998). Muscle blood flow is reduced with dehydration during prolonged exercise in humans. J. Physiol..

[32] Fadel P.J., Keller D.M., Watanabe H., Raven P.B., Thomas G.D. (2004). Noninvasive assessment of sympathetic vasoconstriction in human and rodent skeletal muscle using near-infrared spectroscopy and Doppler ultrasound. J. Appl. Physiol..

[33] Park H.Y., Shin C., Lim K. (2018). Intermittent hypoxic training for 6 weeks in 3000 m hypobaric hypoxia conditions enhances exercise economy and aerobic exercise performance in moderately trained swimmers. Biol. Sport.

[34] Davis S.L., Fadel P.J., Cui J., Thomas G.D., Crandall C.G. (2006). Skin blood flow influences near-infrared spectroscopy-derived measurements of tissue oxygenation during heat stress. J. Appl. Physiol..

[35] Périard J.D., Thompson M.W., Caillaud C., Quaresima V. (2013). Influence of heat stress and exercise intensity on vastus lateralis muscle and prefrontal cortex oxygenation. Eur. J. Appl. Physiol..

